# Baropodometry and stereophotogrammetry for classifying flatfoot severity: dynamic angles and footprint indexes

**DOI:** 10.1186/1757-1146-7-S1-A24

**Published:** 2014-04-08

**Authors:** Claudia Giacomozzi, Paolo Caravaggi, Lisa Berti, Alberto Leardini, Sandro Giannini

**Affiliations:** 1 Department of Technology and Health, Istituto Superiore di Sanità, Rome, Italy; 2 Movement Analysis Laboratory, Istituto Ortopedico Rizzoli, Bologna, Italy; 3 1st Orthopaedic Clinic, Istituto Ortopedico Rizzoli, Bologna, Italy

## Background

Static radiographic angles and other clinical qualitative observations are used traditionally for classifying flatfeet. Correlation of foot shape measurements, taken from pressure footprints under dynamic conditions, with radiographic angles was preliminarily investigated in young flatfeet [1]. The aim of this study is to assess the sensitivity and specificity of existing or purposely defined angles and indexes obtained from both dynamic pressure footprints and multisegment foot kinematics during the stance phase of gait. The main hypothesis is that these thorough measures can account for structural and functional changes in the foot, thus improving flatfoot severity classification.

## Materials and methods

**Figure 1:**
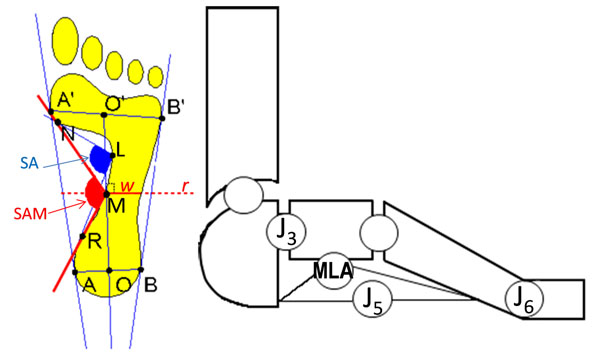
Dynamic footprint indexes which best discriminated between the three groups: SAM (C:105±5°; F1:124±4°; F2:172±13°) and RMFW (C:0.18±0.11; F1:0.42±0.04; F2: 0.69±0.11)

Sixty among healthy volunteers and patients were first assessed clinically and assigned to either Control (C), Level 1 Flatfoot(F1), or Level 2 Flatfoot (F2, more compromised than F1) Groups. Then, data were collected, three consistent trials per foot, on both feet if belonging to different groups, on the right foot only otherwise. A validated integrated pressure-kinematics technique was used based on a VICON motion system, an EMED baropodometer, and the IORfoot model [2]. For this preliminary analysis, 15 patients (10M/5F; BMI 23.0±2.4; age 25.9±7.0 years; stance 670±42.0 ms) were taken, 5 for each group. From each dynamic footprint, the following was calculated (Fig. 1): Subarch Angle (SA) and Arch Index (AI) as in the Novel software; Modified Subarch Angle (SAM) hereby defined as originated at point M rather than L; Midfoot/Forefoot Ratio (RMFW) hereby defined as the ratio between *w* and A’B’. Sagittal plane ROM for foot joints J3, J5, J6 and for medial longitudinal arch (MLA), frontal ROM for J3 and J6, and the absolute value at midstance for MLA were also calculated.

## Results

**Figure 2:**
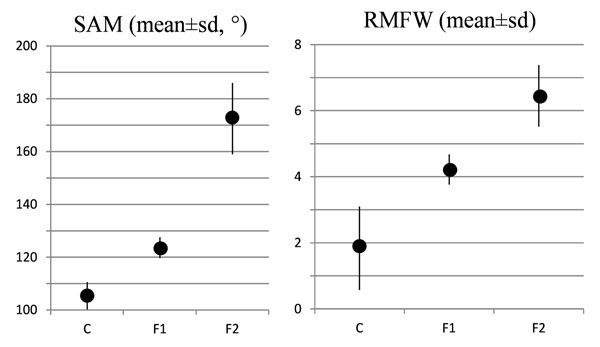
On the left: dynamic footprint measurements as in the Novel software. Additionally: M represents the intersection between medial midfoot and the line r perpendicular to the bisecting line through OO’ midpoint; *w* is the midfoot width over line r. On the right: joints of the IOR foot model, and MLA angle .

The three five-subject groups were found homogeneous as for BMI, age and stance duration. SAM (C:105±5°; F1:124±4°; F2:172±13°) and RMFW (C:0.18±0.11; F1:0.42±0.04; F2: 0.69±0.11) best discriminated among the three groups (Fig. 2), without any overlapping. AI was more variable in the C group (0.17±0.08) and did not discriminate well between C and F1, as well as SA and MLA; J3, J5 and J6 showed non-statistically significant differences among the three groups.

## Conclusions

Sensitivity and specificity will be more thoroughly estimated on the whole dataset of the 60 examined individuals. Preliminarily, SMA and RMFW seem to be the most appropriate dynamic footprint indexes for classifying flatfeet. MLA angle at midstance seems to be specific for F2 only, accounting for significant structural changes with respect to C and F1. MLA angle ROM might help to distinguish, within the same pathologic group, between flexible and rigid flatfoot.
